# Proteomics Reveals How the Tardigrade Damage Suppressor Protein Teaches Transfected Human Cells to Survive UV-C Stress

**DOI:** 10.3390/ijms241411463

**Published:** 2023-07-14

**Authors:** Enxhi Shaba, Claudia Landi, Carlotta Marzocchi, Lorenza Vantaggiato, Luca Bini, Claudia Ricci, Silvia Cantara

**Affiliations:** 1 Functional Proteomics Lab, Department of Life Sciences, University of Siena, 53100 Siena, Italy; enxhi.shaba@unisi.it (E.S.); lorenz.vantaggiato2@unisi.it (L.V.); luca.bini@unisi.it (L.B.); 2 Department of Medical, Surgical and Neurological Sciences, University of Siena, 53100 Siena, Italy; carlottamarzocchi@libero.it (C.M.); claudia.ricci@unisi.it (C.R.); cantara@unisi.it (S.C.)

**Keywords:** Dsup, 2DE, DNA damage response, stress granules, telomere length, mRNA stability

## Abstract

The genome sequencing of the tardigrade *Ramazzottius varieornatus* revealed a unique nucleosome-binding protein named damage suppressor (Dsup), which was discovered to be crucial for the extraordinary abilities of tardigrades in surviving extreme stresses, such as UV. Evidence in Dsup-transfected human cells suggests that Dsup mediates an overall response in DNA damage signaling, DNA repair, and cell cycle regulation, resulting in an acquired resistance to stress. Given these promising outcomes, our study attempts to provide a wider comprehension of the molecular mechanisms modulated by Dsup in human cells and to explore the Dsup-activated molecular pathways under stress. We performed a differential proteomic analysis of Dsup-transfected and control human cells under basal conditions and at 24 h recovery after exposure to UV-C. We demonstrate via enrichment and network analyses, for the first time, that even in the absence of external stimuli, and more significantly, after stress, Dsup activates mechanisms involved with the unfolded protein response, the mRNA processing and stability, cytoplasmic stress granules, the DNA damage response, and the telomere maintenance. In conclusion, our results shed new light on Dsup-mediated protective mechanisms and increases our knowledge of the molecular machineries of extraordinary protection against UV-C stress.

## 1. Introduction

The damage suppressor (Dsup) is a unique nucleosome-binding protein that was discovered for the first time in 2016 when scientists completed the sequencing of the *Ramazzottius varieornatus* tardigrade genome [1]. Around 1464 species of tardigrades are found worldwide to date [2], and they are considered aquatic because they require a layer of water around their bodies to prevent dehydration. Tardigrades have been observed from the deep sea to sand dunes, and they are able to survive extreme environments, including space. In particular, X-ray protection seems to be exerted by Dsup thanks to a direct bind to chromatin [3], but tardigrades can also tolerate increased temperatures and pressure [4,5], as well as oxidative stress and dehydration [6]. Recently, Dsup has been gaining great interest among the scientific community. In particular, human and plant cells have been engineered to produce Dsup, resulting in the acquisition of a higher resistance to X-rays [1], oxidative stress [3], and genomutagens [7]. Recently, it has been demonstrated that Dsup affects the expression of endogenous genes under stress conditions, such as UV-C exposure, in human-transfected cells. Particularly, mRNA evidence showed that the expression of genes involved in DNA repair and cell cycle checkpoints is upregulated together with transcription factor modulation [8]. According to their extraordinary potential, tardigrades may represent a great chance of exploring new ways of adaptation to surrounding environments. In fact, as commonly accepted, our planet is facing massive changes, principally due to human abuse and unlimited exploitation of the Earth’s resources. These events are leading to the extensive alteration of macro- and micro-environments such as drought and weather changes; therefore, the exposure to these phenomena is often unbearable for common-living organisms from plants to humans [9]. UV radiation may be classified into three primary types: ultraviolet A (UV-A, 315–399 nm); ultraviolet B (UV-B, 280–314 nm); and ultraviolet C (UV-C, 100–279 nm). UV-A is not filtered by the ozone layer whereas UV-B is only absorbed partially. Even if UV-C is completely absorbed by the ozone layer and atmosphere, they represent the most damaging radiation type as the lower the wavelength, the higher the energy content in UV radiation. Specifically, they cause cyclobutane pyrimidine dimers (CPDs) and pyrimidine-6,4-pyrimidinone photoproducts (6,4PPs) on exposed DNA, as well as UV-associated ROS production via photodynamic reactions exerting high DNA-damaging effects [10]. Both CPD and 6,4PP lesions alter DNA structure by introducing bends that inhibit cell transcription and replication. After the replicative arrest, cells undergo a progressive telomere erosion which contributes to genetic instability and accelerates cell death by apoptosis [11,12]. In addition, telomeres are hypersensitive to single-stranded DNA damage; therefore, their protection is essential for cell life and DNA stability. Given these increasing and damaging risks, the strong necessity for exploring new ways of adapting to these newly forming environmental scenarios, as tardigrades do, suits perfectly well with high-throughput technologies such as OMIC sciences. In fact, these approaches are able to provide a wider perspective and a more comprehensive knowledge of biological complexity at multiple levels.

To this purpose, we applied a functional proteomic approach investigating the molecular mechanisms modulated by Dsup in transfected human cells and exploring the Dsup-activated molecular pathways under UV-C stress. In particular, we performed a differential proteomic analysis of Dsup-transfected (Dsup+) and control (Dsup−) HEK293T cells under basal conditions and at 24 h recovery after 15 s of exposure to UV-C. Various molecular pathways and differential proteins of interest were tested to confirm their modulation in the presence of Dsup at a cellular level.

## 2. Results

### 2.1. In the Absence of External Stimuli, Dsup-Transfected Cells Present a Modulation of Proteins Involved in Protein Folding, Telomere Maintenance, and Metabolic Processes

**Figure 1:**
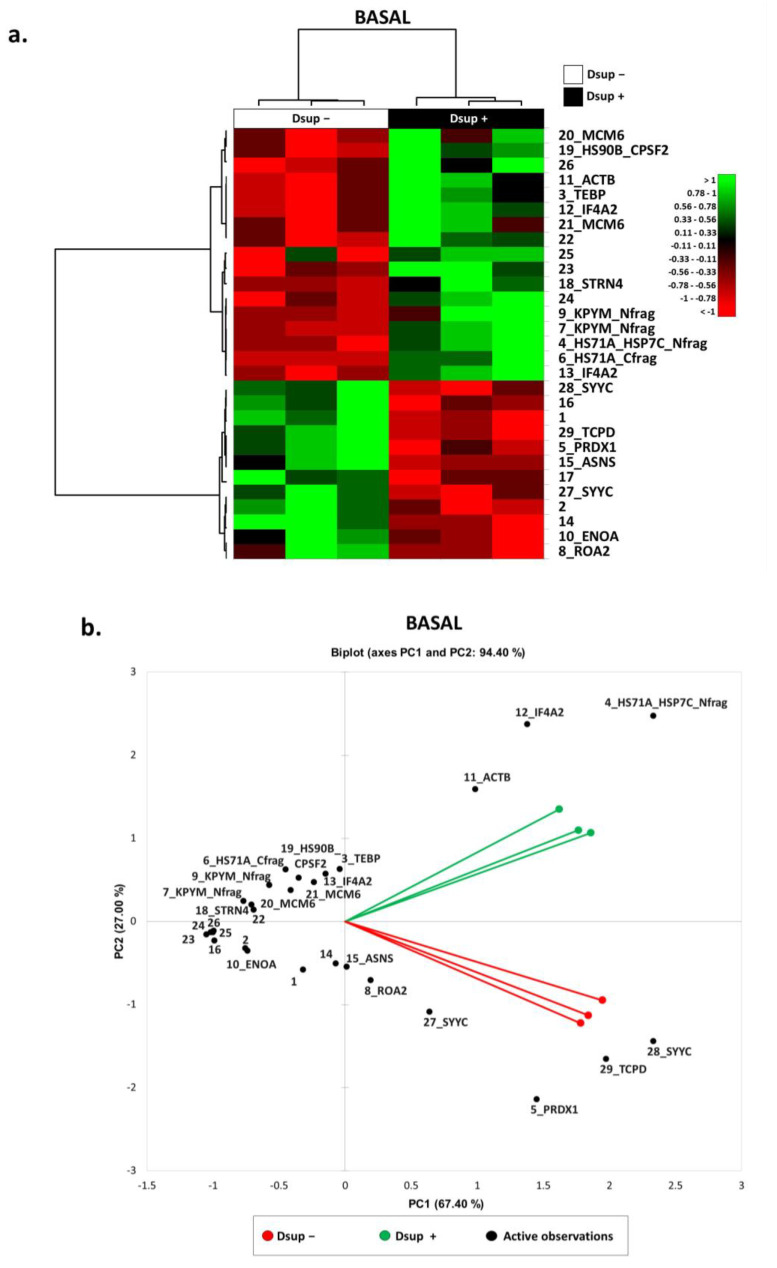
(**a**) Heatmap analysis of the differential spots found via proteomic analysis of Dsup− vs. Dsup+ HEK293T cell cultures at basal condition. As shown in the legend, high-abundance spots are reported in green while low-abundance ones are reported in red. Black numbers and acronyms represent differential protein spots, while Dsup− cell cultures are reported in black, and Dsup+ cell cultures are reported in white. (**b**) Principal Component Analysis of the differential spots found via proteomic analysis of Dsup− vs. Dsup+ HEK293T cell cultures at basal condition. PCA summarizes a 94% of variance (PC1: 67.40% and PC2: 27%). Black numbers and acronyms represent differential protein spots, while Dsup− cell cultures are reported in red and Dsup+ cell cultures are reported in green.

In order to understand whether Dsup itself was able to activate cellular mechanisms in the absence of external stresses, we applied a differential proteomic analysis between Dsup− and Dsup+ cells at basal conditions. About 3800 spots per gel were detected using image analysis software. We highlighted 29 differentially abundant spots (Table S1 and in Figure S1), of which 19 were identified using MALDI-ToF. As reported by the heatmap analysis in Figure 1a, 17 were higher abundant and 12 were lower abundant after Dsup transfection. In particular, the PCA (Figure 1b) shows that Dsup− and Dsup+ proteomic profiles are well-separated from each other, highlighting the Dsup impact in the cellular proteome. Based on the PCA, the increased abundance of eukaryotic initiation factor 4A-II (IF4A2), actin, cytoplasmic 1 (ACTB), and heat shock 70 kDa protein 1A N-term fragment (HS71A_HS71C N-frag), and the decreased abundance of asparagine synthetase (glutamine-hydrolyzing) (ASNS), heterogeneous nuclear ribonucleoproteins A2/B1 (ROA2), peroxiredoxin 1 (PRDX1), T-complex protein 1 subunit delta (TCPD), tyrosine-tRNA ligase, and cytoplasmic (SYYC) have a greater relevance in Dsup−/+ cell-culture discrimination.

**Figure 2:**
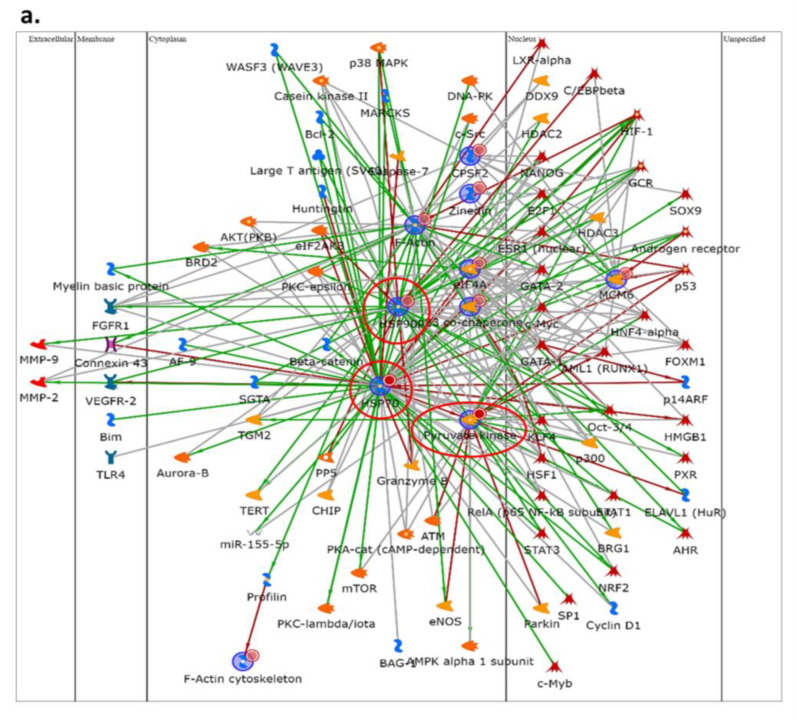

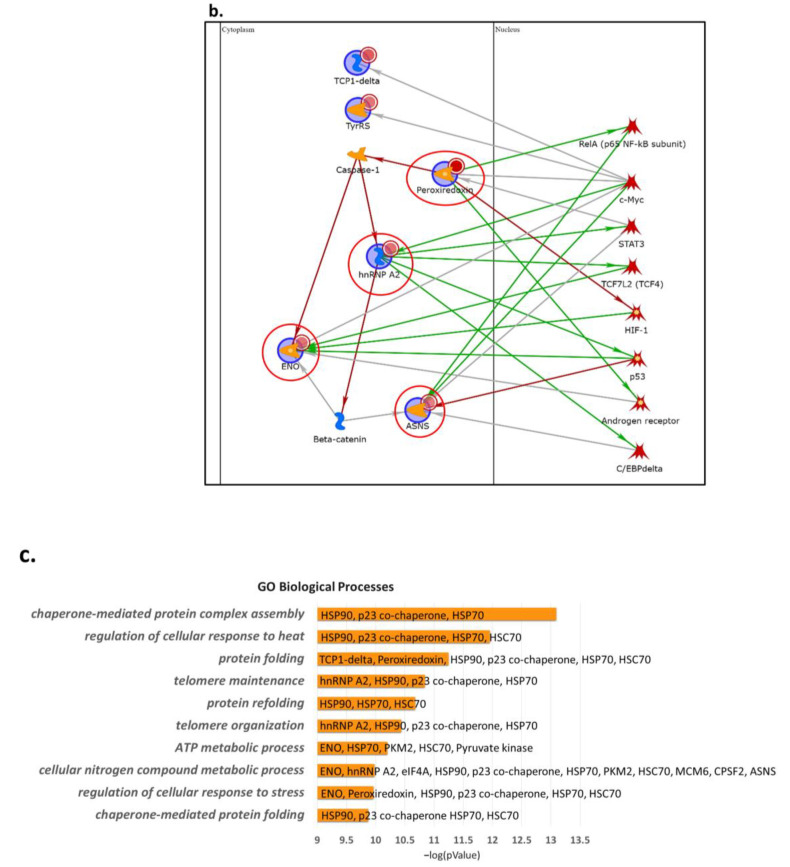
Network analysis using MetaCore software to highlight characteristic protein interactions of the higher- (**a**) and lower-abundance (**b**) differential protein species of Dsup− vs. Dsup+ HEK293T cell cultures analysis at basal condition. (**a**) HSC70, HSP90 beta, PKM2, and HSP90 are the central functional hubs of the net and are marked in red circles. (**b**) ENO1, ASNS, ROA2, PRDX1, and TCPD are the central functional hubs, marked in red circles. (**c**) Enrichment analysis via GO Biological Processes using MetaCore software: each histogram represents an enriched GO Biological Process associated to its *p*-value in −log10 and its related differential proteins.

Figure 2a shows the protein network of the differentially higher-abundance proteins in Dsup+ basal cells with HSP70, HSP90, and PKM2 acting as central functional hubs, suggesting their pivotal role in downstream or upstream molecular pathway regulation following Dsup transfection. On the other hand, Figure 2b reports the protein network of the differentially lower-abundance proteins in basal cells after Dsup transfection, showing that ENO, ASNS, ROA2, PRDX1, and TCPD are, in this case, central functional hubs. In addition, an enrichment analysis of GO Biological Processes of all differential proteins in Dsup−/+ cells was performed (Figure 2c). Our results highlight that the differential proteome of Dsup+ cells is characterized by a modulation of proteins involved in a chaperone-mediated protein complex assembly (HSP90, p23 co-chaperone, and HSP70); protein folding and refolding (TCP1-delta, peroxiredoxin, HSP90, p23 co-chaperone, HSP70, and HSC70); telomere maintenance and organization (hnRNP A2, HSP90, p23 co-chaperone, and HSP70); ATP metabolic processes (ENO, HSP70, PKM2, HSC70, and Pyruvate kinase); cellular nitrogen compound metabolic processes (ENO, hnRNP A2, eIF4A, HSP90, p23 co-chaperone, HSP70, PKM2, HSC70, MCM6, CPSF2, and ASNS); and the regulation of cellular response to stress (ENO, Peroxiredoxin, HSP90, p23 co-chaperone, HSP70, and HSC70).

**Figure 3:**
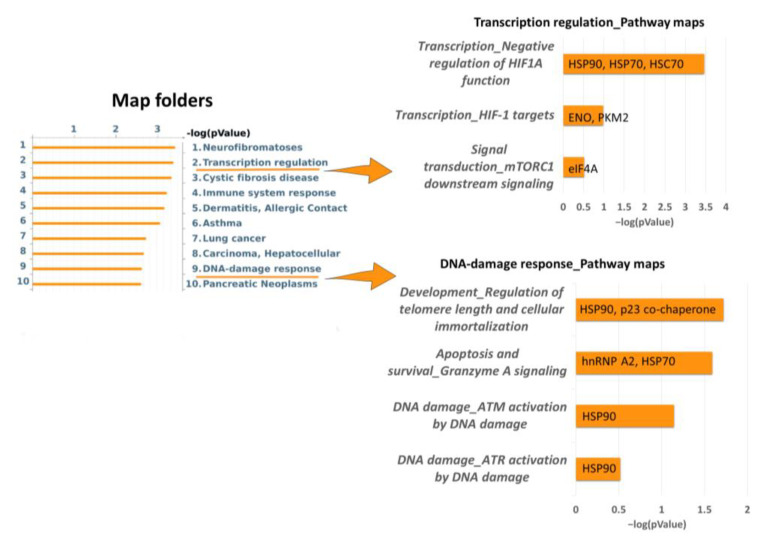
Enrichment analysis via map folder analysis using MetaCore software, performed with all differential proteins of Dsup- vs. Dsup+ proteomic analysis (basal condition). Figure 3 also reports Pathway maps in Folder ‘Transcription regulation’ and Pathway maps in Folder ‘DNA-damage response’. Each histogram represents an enriched map folder/Pathway map associated with its *p*-value in −log10 and its related differential proteins.

Moreover, enrichment analysis via map folders (Figure 3) evidenced transcription regulation and DNA-damage response as two important molecular mechanisms regulated via identified proteins. In particular, transcription regulation is related to the negative regulation of the HIF1α function and HIF-1 targets (HSP90, HSP70, HSC70, ENO, and PKM2), in addition to mTORC1 downstream signaling (eIF4A); whereas the DNA-damage response is related to the regulation of telomere length (HSP90 and TEBP/p23 co-chaperone), apoptosis, and survival using granzyme A signaling (ROA2, HSP70), ATM, and ATR (Figure 3).

### 2.2. Proteins Involved in DNA-Damage Response and in the Regulation of Transcription Are Increased in Dsup+ Cells after 15″ of UV-C Irradiation Followed by 24 h of Recovery

**Figure 4:**
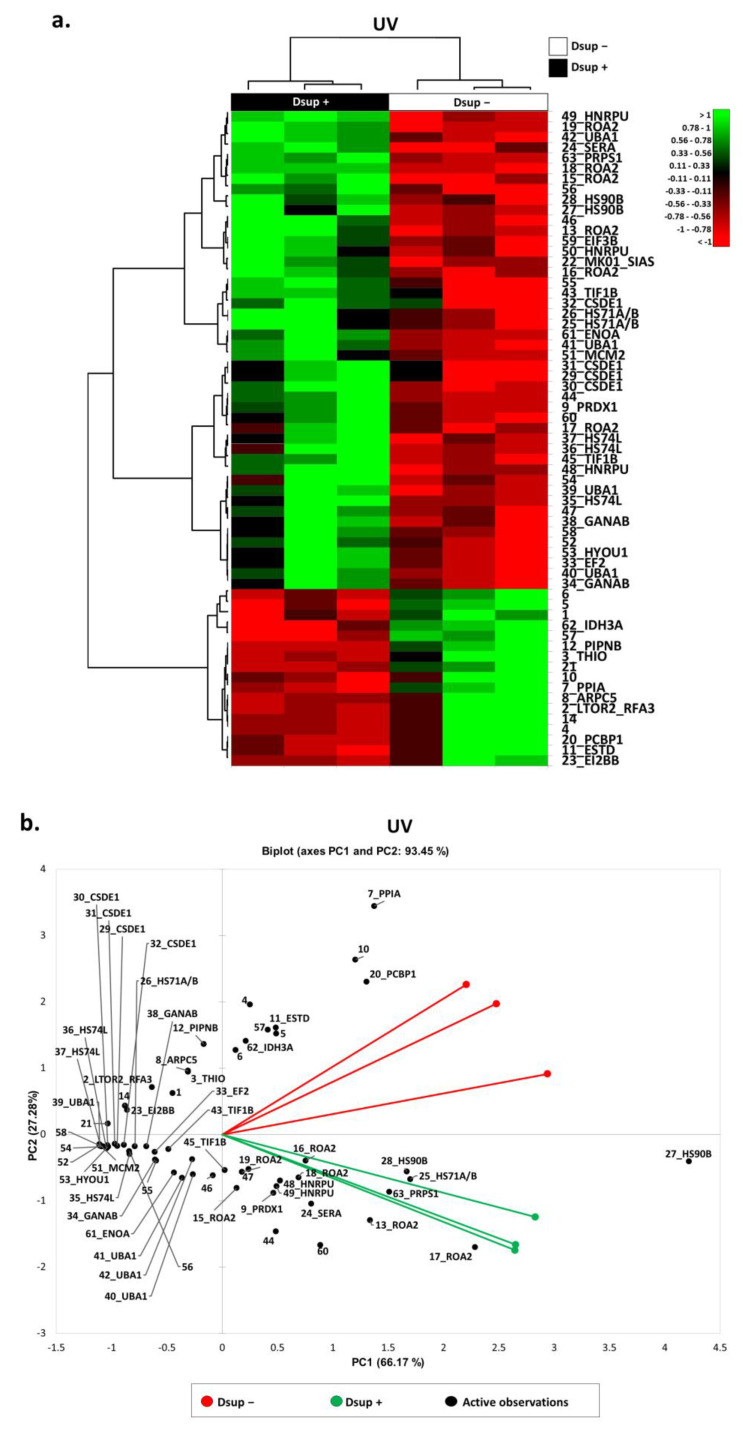
(**a**) Heatmap analysis of the differential spots found via proteomic analysis of Dsup− vs. Dsup+ HEK293T cell cultures at 24 h recovery after 15 s of UV-C irradiation. As shown in the legend, high-abundance spots are reported in green while low-abundance ones are reported in red. Black numbers and acronyms represent differential protein spots, while Dsup− cell cultures are reported in white and Dsup+ cell cultures are reported in black. (**b**) Principal Component Analysis of the differential spots found by proteomic analysis of Dsup− vs. Dsup+ HEK293T cell cultures at 24 h recovery after 15 s of UV-C irradiation. PCA summarizes 93.45% of variance subdivided for 66.17% in PC1 and 27.28% in PC2. Black numbers and acronyms represent differential protein spots, while Dsup− cell cultures are reported in red and Dsup+ cell cultures are reported in green.

Our group already demonstrated that Dsup+ cells that are exposed to UV-C and that allowed for recovery for 24 h presented a statistically significant increase in survival rate compared to Dsup− (Figure S2) with the activation of transcription factors and overexpression of ATM and ATR mRNAs [8]. Considering these previous data, we aimed to investigate deeper into which cellular mechanisms might be activated by Dsup to promote cell survival after UV-C radiation at protein level. Similarly, to discern which performed in basal conditions, we performed a differential proteomic analysis of Dsup−/+ cells exposed to UV-C. About 3900 spots per gel were detected using image analysis software. We highlighted 63 differentially abundant spots (Table S2 and Figure S3), of which 46 were identified via MALDI-ToF. The heatmap in Figure 4a shows that 46 spots were differentially more abundant while 17 spots were differentially less abundant after Dsup transfection. Moreover, Figure 4b reports the PCA of Dsup−/+ cells in a 2D spatial distribution, showing that Dsup significantly impacts the differential proteome after UV-C irradiation with a higher extent compared to basal conditions. Particularly, it also highlighted that the decreased amounts of PPIA, PCBP1, ESTD, IDH3A, PIPNB, ARPC5, THIO, LTOR2/RFA3, and EI2BB strongly influenced the Dsup− cells’ clustering, whereas the increased amounts of HS90B, ROA2, SERA, PRPS1, HS71A/B, HNRPU, PRDX1, and TIF1B considerably influenced the Dsup+ cells’ one.

**Figure 5:**
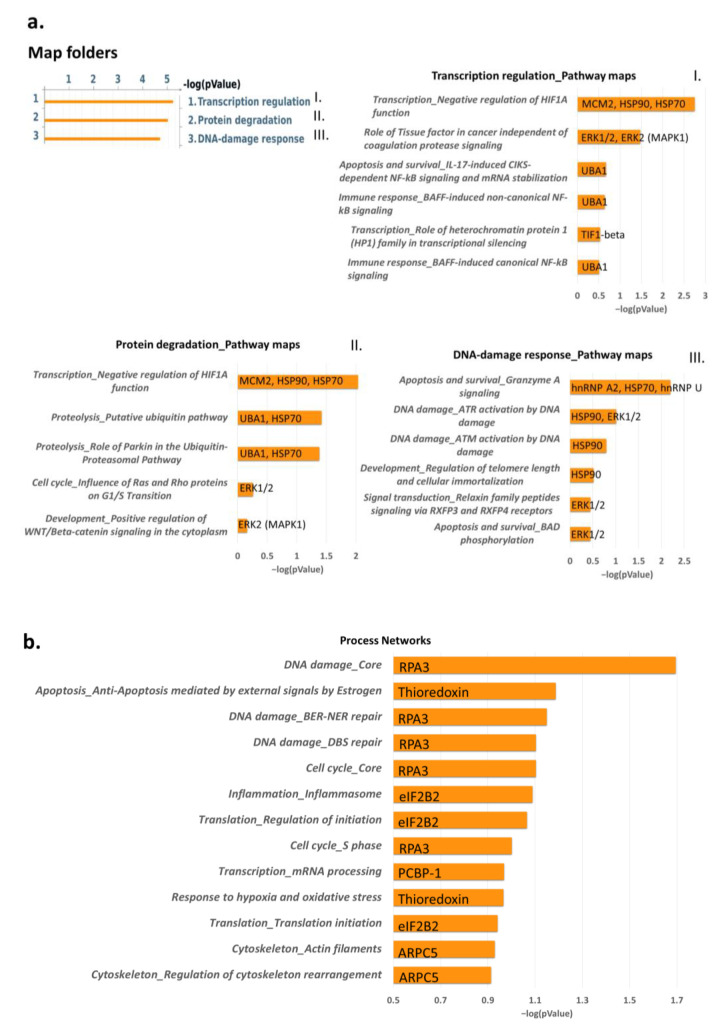
(**a**) Enrichment analysis through map folder analysis using MetaCore software, performed with the differentially higher abundant proteins in Dsup+ HEK293T cells at 24 h recovery after 15 s of UV-C irradiation. Figure 5 also reports Pathway maps in Folder ‘Transcription regulation’ (I), in Folder ‘Protein degradation’ (II), and in Folder ‘DNA-damage response’ (III). (**b**) Enrichment analysis via process networks analysis using MetaCore software, performed with the differentially low-abundance proteins in Dsup+ HEK293T cells at 24 h recovery after 15 s of UV-C irradiation. Each histogram represents an enriched process network associated with its *p*-value in −log10 and its related differential proteins.

An enrichment analysis via map folders of differentially higher abundant proteins in Dsup+ cells is reported in Figure 5a, showing three relevant molecular pathways where each grouping of particular mechanisms is associated with the identified higher-abundance proteins: transcription regulation, protein degradation, and DNA-damage response. Figure 5a(I) shows specific pathways related to transcription regulation: we put in evidence that the higher abundance of the two protein species, HSP90 (spots 27 and 28) and HSP70 (spot 25 and 26), together with the higher amount of DNA replication licensing factor (MCM2; spot 51), was implicated with a negative regulation of HIF1A. We also found the higher abundance of four proteoforms of a ubiquitin-like modifier-activating enzyme 1 (UBA1; spot 39–42), as well as the involvement of two protein species of transcription intermediary factor 1-beta (TIF1B and TIF1-beta; spots 43, 45) involved in NF-kB signaling-related processes and in the role of heterochromatin protein 1 family in transcriptional silencing, respectively. Secondly, Figure 5a(II) reports additional pathways associated with protein degradation, such as ubiquitin-proteasomal pathways, which are suggested by the higher amounts of HSP70 and UBA1, as well as due to the signaling transduction processes advised by the more abundant mitogen-activated protein kinase 1 (MK01 and ERK1/2; spot 22). Furthermore, Figure 5a(III) evidences particular pathways related to the DNA-damage response (DDR), such as ATM/ATR activation by DNA damage and regulation of telomere length and cellular immortalization associated with the higher amounts of HSP90 and MK01.

On the other hand, an enrichment analysis via process networks of differentially lower-abundance proteins in Dsup+ cells is reported in Figure 5b. Particularly, DNA-damage response processes, including base excision repair (BER)/nucleotide excision repair (NER) events, double-stranded DNA break (DSB) repair, and cell-cycle-regulatory processes are reported in association with the replication protein A 14 kDa subunit (RFA3 and RPA3; spot 2). Transcriptional regulatory mechanisms of mRNA processing and translation are associated with the lower abundance of the translation initiation factor eIF2B subunit beta (EI2BB and eIF2B2; spot 23). Other relevant pathways are the response to hypoxia and oxidative stress (THIO; spot 3), as well as the regulation of cytoskeleton rearrangement indicated by the lower amount of actin-related protein 2/3 complex subunit 5 (ARPC5; spot 8).

**Figure 6:**
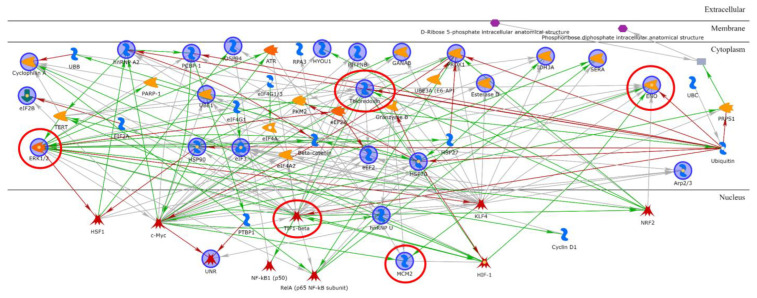
Network analysis using MetaCore software to highlight characteristic protein interactions of all the differential protein species of the proteomic analysis of Dsup− vs. Dsup+ HEK293T cell cultures at 24 h recovery after 15 s of UV-C irradiation. Thioredoxin, ERK1/2, TIF1-beta, MCM2, and ENOA are the central functional hubs of the net and are marked in red circles.

In order to explore potential interactions among the identified proteins, a network analysis was performed (Figure 6) where TIF1-beta, ERK1/2, ENO1, Thioredoxin, and MCM2 were reported to be central functional hubs. In particular, TIF1-beta showed several interactions with other differentially abundant proteins, such as peptidyl-prolyl cis-trans isomerase A (PPIA and cyclophilin A; spot 7); eukaryotic initiation factor 4AII (IF4A2 and eIF4A; spot 12, 13); eukaryotic translation initiation factor 3 subunit B (EIF3B and eIF3; spot 59); and elongation factor 2 (EF2 and eEF2; spot 33). In addition, MCM2 displayed interactions with ATR serine/threonine protein kinase, HIF-1, and c-Myc, which were reported to be connected to heterogeneous nuclear ribonucleoproteins A2/B1 (ROA2 and hnRNP A2; spot 13, 15–19) and U (hnRNP U; spot 48–50), as well as the cold shock domain-containing protein E1 (CSDE1 and UNR; spot 29–32).

### 2.3. Targeted Validation of Proteins Belonging to Unfolded Protein Response (UPR), Transcription and Metabolic Regulation, DDR, and Telomere Length Regulation Pathways

**Figure 7:**
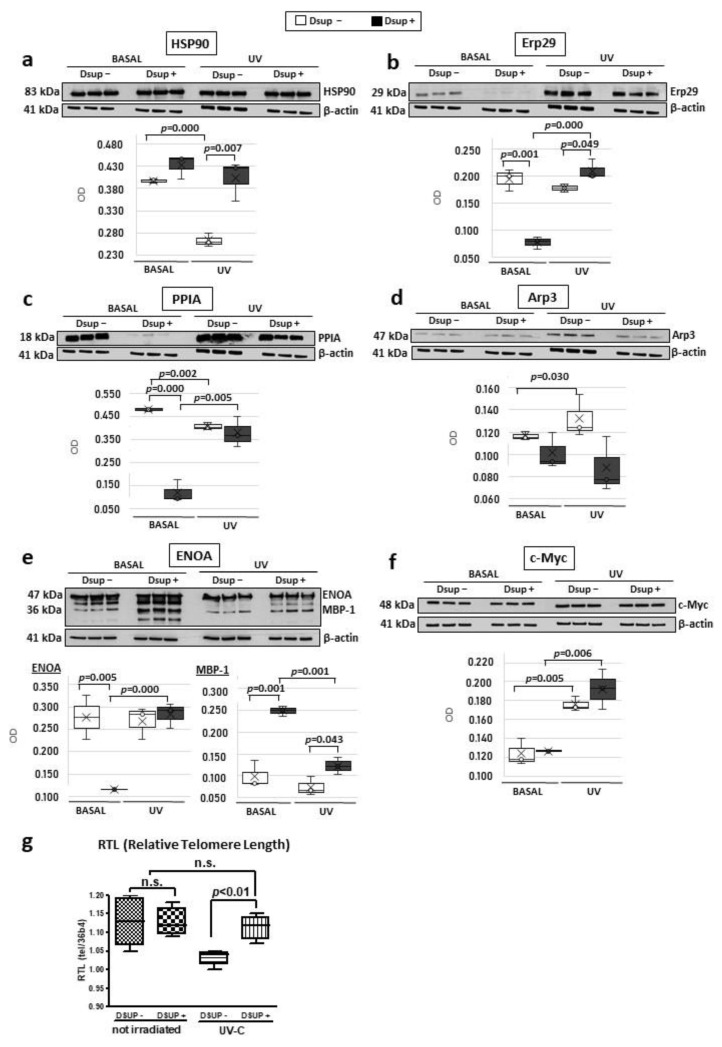
Western blot validations and relative telomere length (RTL) assay. Figure reports WB validations of HSP90, (**a**) Erp29, (**b**) PPIA, (**c**) Arp3, (**d**) ENOA and MBP-1, (**e**), and c-Myc, (**f**) and normalization of bands’ intensity was performed on total β-actin. Legend displays that normalized WB lane intensities of Dsup cells are reported as white, while those of Dsup + cells are reported in black. Differences are considered significant with *p*-value < 0.05. Relative telomere length (RTL) assay (**g**) displays the measurement via real-time PCR of the relative telomere length in Dsup− cells compared to Dsup+ cells exposed to UV-C. *p* < 0.01 via one-way ANOVA with Fisher’s correction.

Given the differential proteomic data and the specific molecular pathways suggested via bioinformatic analysis, a targeted validation was performed. HSP90 is a differential protein found more abundant in Dsup+ cells in basal conditions and after UV-C irradiation. As a central factor in protein-folding processes, as well as transcription regulation, DNA repair, and regulation of metabolic processes following cell stress [13], Dsup-mediated HSP90-increased abundance might be a crucial effector of Dsup protection. As shown in Figure 7a, Western blot analysis confirms a moderate increase in abundance in Dsup+ cells at the basal level. Interestingly, HSP90 abundance in Dsup− cells drastically decreases after UV-C irradiation while HSP90 levels remain almost unchanged between basal and irradiated conditions in Dsup+ cells, thus showing a significant increase after UV-C irradiation compared to the Dsup− counterpart.

Since UPR is a molecular process highlighted in our proteomic results, we evaluated Erp29 protein levels. Erp29 abundance, indeed, increases in response to endoplasmic reticulum stress (ERS) [14], especially in the case of genotoxic stress induced by ionizing radiation [15]. As shown in Figure 7b, Erp29 significantly decreases in Dsup+ cells at the basal level and then statistically increases after UV-C irradiation in comparison to Dsup− cells. Indeed, Dsup− cells show a decreased level of Erp29 after UV-C treatment.

Since PPIA is a multifunctional protein that acts also in UPR, we performed a Western blot assay which highlights a significant decreased abundance of PPIA in Dsup+ basal cells in addition to a significant decrease in Dsup− cells and a significant increase in Dsup+ cells after UV-C irradiation, compared to their basal counterpart (Figure 7c). ARPC5, which we found lowers abundance in Dsup+ cells after UV-C stress, is a subunit of the Arp2/3 complex [16]. Therefore, we decided to perform a Western blot assay for Arp3 to verify if another member of the complex is modulated by Dsup transfection, since the complex promotes actin polymerization in the nucleus regulating gene transcription and DNA-damage repair [17]. As expected, Arp3 is less abundant in Dsup+ cells at the basal level and after UV-C stress, with respect to the corresponding Dsup− cells displaying the same trend in ARPC5 and suggesting that the entire complex is less abundant in Dsup+ cells (Figure 7d).

Notably, proteomic data also suggest a cellular metabolic modulation in Dsup+ cells, as indicated by the dysregulation of different protein species of ENOA. Figure 7e highlights an interesting trend of ENOA proteoforms validated using the Western blot. In basal conditions, Dsup+ cells present various proteoforms at a lower molecular weight than the referring protein at 47 kDa, which results in significantly increased abundance in Dsup+ cells and confirms proteomic data. There is a particular ENOA proteoform (37kDa), referred to as c-Myc Binding Protein 1 (MBP-1) [18] and missing of the first 90 amminoacids. This proteoform appears to be significantly higher abundant in Dsup+ basal and UV-C irradiated cells, compared to the Dsup− counterparts. This proteoform is reported to inhibit the expression of c-Myc, which is, in turn, statistically increased at protein level in both Dsup− and Dsup+ cells after UV-C stress (with a higher extent for Dsup+ cells, such as in Figure 7f).

Since proteomics highlights proteins involved in telomere maintenance, we investigated telomere stability in Dsup− and Dsup+ cells after UV-C irradiation by measuring the relative telomere length (RTL) by qPCR. As shown in Figure 7g, RTL is significantly longer in Dsup+ cells compared to Dsup− cells at 24 h recovery after UV-C exposure (*p* < 0.01). No differences in RTL were found in non-irradiated cells and between non-irradiated cells and Dsup+ cells after UV-C exposure (Figure 7g).

## 3. Discussion

The Dsup protein was reported for the first time by Hashimoto T. et al. as a prominent example of tardigrade-unique abundant proteins involved in tolerability, as it resulted in a DNA-associating protein mediating DNA protection against radiation in cultured animal and human cells [1]. For these reasons, our study focused on applying a functional proteomic approach aimed at investigating Dsup impact in human cells in basal conditions and after UV-C exposure and investigating the potentially modulated mechanisms in response to induced stress.

In this study, we evidenced for the first time that, even at basal conditions, the Dsup protein impacts cells by modulating proteins associated with the UPR. Indeed, at basal conditions, we highlighted several differentially abundant proteins belonging to the protein folding, telomere maintenance, and metabolic process pathways. One of the main players we found to be highly abundant in Dsup+ transfected-human cells is HSP90, which is reported to significantly mediate cellular resistance via UPR pathway [19]. Our results also report the differential abundance of ENOA, which is reported to act as a glycolytic enzyme, a plasminogen receptor, and a DNA-binding protein [20]. Moreover, it is reported to be involved in protective processes by enhancing cell viability and glycolysis against various stresses [21,22,23,24]. In our study, ENOA activation was particularly evident after UV-C exposure. Interestingly, ENOA presents several proteoforms that are related to multiple functions and significance [24]. Of notice, we detected the increased amount of a 37kDa proteoform in Dsup+ cells, which could be the myc promoter-binding protein 1 (MBP-1), a shorter protein variant of ENOA involved in the c-myc regulation [18] and reported to have increased as a consequence of cell response to stress conditions [25].

As a result of UV exposure, there is the formation of helix-distorting lesions, such as bulky DNA adducts (CPDs and 6,4PPs), which interfere with DNA transcription and replication and lead also to the production of double-stranded breaks (DSBs) [26]. DSBs might also occur in UV-C irradiated cells as a consequence of single-stranded breaks (SSBs) and repair failure via base excision repair (BER) [27,28]. In addition, UV light might also indirectly cause DNA damage via ROS production in photodynamic reactions, leading to ROS-damaged bases and direct SSBs [10]. The occurrence of these lesions canonically triggers the DDR via ATR and/or ATM activation which initiates signaling pathways of DNA-damage sensing and repair. The main activated DNA-repair mechanism following UV-induced DNA damages is nucleotide excision repair (NER) [29]. Interestingly, our results indicate a higher abundance of one protein species of ERK1/2 in Dsup+ UV-C stressed cells and especially ERK1/2 is associated with the DDR signaling pathway via an enrichment analysis. Indeed, the activation of ERK1/2 has been reported to be activated via UV exposure [30] and to potentially modulate nucleotide excision repair (NER) pathways [31]. Moreover, our functional proteomic analysis reports a higher abundance of two protein species of TIF1B (TIF1B; TRIM28; and KAP1) in Dsup+ UV-C stressed cells, which is reported to be involved in DNA-damage repair mechanisms, also following UV exposure [32,33]. The network analysis suggests that TIF1B represents a functional hub interacting with several differential proteins in Dsup+ UV-C stressed cells. Among these, it regulates the transcription of ARPC5 (the subunit of the Arp2/3 complex) [34], which supports actin polymerization in the nucleus and contributes to the gene transcription and DNA-repair processes [35]. In Dsup+ cells, we observed a concomitantly lower abundance of Arp3 and the replication factor A 14 kDa subunit (RPA3), the latter being the smallest subunit of the RPA complex which is an essential coordinator of DNA repair [36]. Moreover, the network analysis also reports that TIF1B transcriptionally regulates UBA1 [37] and we observed a higher abundance in four proteoforms of UBA1 in Dsup+ cells after UV-C exposure. Indeed, the ubiquitination pathway has a positive regulatory role for efficient NER machinery, potentially recruiting repair factors to DNA-damage sites and UBA1 is reported to be potentially involved in the repair of UV-induced DNA damage [38]. In addition, the network analysis also suggests that in Dsup+ UV-C stressed cells, TIF1B interacts with telomerase reverse transcriptase (TERT), as previously reported by Agarwal N. et al., who describes that TIF1B phosphorylation by mTORC1 allows it to induce TERT transcription [39]. The telomere maintenance mechanisms might be crucial events in Dsup+ cells, as supported by the dysregulation of several other differential proteins such as Prostaglandin E synthase 3 (TEBP/p23 co-chaperone), HSP90, HNRPU, and ROA2 [40,41,42,43]. Our functional analysis further suggests a potential interaction between TERT and PPIA [44], which is decreased in abundance in Dsup+ cells exposed to UV-C and implicated in the regulation of cellular processes, such as protein folding, transcriptional regulation, cell survival, and response to stress [45]. Of interest, Daneri-Becerra et al. report PPIA to be a mitochondrial factor which complexes with HSP90 and p23 co-chaperone and plays a significant role in cell survival upon stress [46]. Moreover, PPIA is also reported as a functional interacting partner of the TAR DNA-binding protein (TARDBP or TDP-43), which is predominantly an RNA/DNA-binding protein involved in RNA processing and metabolism, including RNA transcription, splicing, transport, and stability. Specifically, TARDBP is a key component in hnRNP complexes, in which hnRNP A2/B1 is the major heterogeneous ribonucleoprotein recognized by PPIA and is essential in regulating the maturation of newly formed nuclear RNAs/pre-mRNAs into messenger RNAs (mRNAs) [47,48,49,50]. Interestingly, in Dsup+ cells exposed to UV-C, we report the increased abundance of numerous proteoforms of heterogeneous nuclear ribonucleoproteins A2/B1 (ROA2) in addition to heterogeneous nuclear ribonucleoprotein U (hnRPU). In light of these findings, Dsup seems to have a strong impact on the modulation of mRNA stability processes, as also suggested by the downregulation in Dsup+ cells of Poly(rC)-binding protein 1 (PCBP1), a specific RNA-binding protein that associates with cytoplasmic polyadenylation elements contributing to mRNA stability and translational activity [51].

Cellular responses to environmental stresses often target fine regulation of the transcriptome by activating a set of conserved processes aimed at restoring cellular homeostasis side-by-side with the UPR. One crucial event is represented by the formation of membraneless compartments, referred to as cytoplasmic stress granules (SGs), especially composed of untranslated mRNAs, translation initiation factors, small ribosomal subunits, and RNA-binding proteins (RBPs). Their main advantages are reduced energy consumption, regulation of proteostasis, and cell survival improvement under damaging conditions [52]. Many differentially higher-abundance proteins in Dsup+ cells are associated with SGs, such as the four proteoforms of CSDE1 [53,54], EIF3B, IF4A2, and ROA2. In detail, IF4A2 (the component of the eIF4F complex) is required for the so-called “non-canonical” stress granule formation that, among its components, also includes EIF3B (the component of the eIF3 complex) and ROA2 (as part of the required hnRNPs) [55]. Of note, this stress response is only transient, leading SGs to be disassembled when the insult is removed and the translation is restored. Recently, SGs’ definition has been evolving into dynamic cytoplasmic biocondensates in which intrinsically disordered proteins (IDPs) are essential for the assembly [56]. IDPs are characterized by a lack of persistent tertiary and/or secondary structure, making them extremely dynamic and flexible [57]. Dsup has been recognized as an IDP [58]. The potential involvement of IDPs in the formation of other protective structures, like SGs, in response to diverse stresses might potentially suggest further tardigrade-adaptive mechanisms [59] and broaden the Dsup-mediated protective impact in human cells.

## 4. Materials and Methods

### 4.1. Cell Cultures, Dsup Transfection, and UV-C Radiation

The HEK293T cell line was kindly donated by Prof. Sandra Donnini (University of Siena). The HEK293T cells (mycoplasma-free, verified by N-GARDE Mycoplasma PCR reagent set, Euroclone) were maintained in Dulbecco’s Modified Eagle’s Medium (DMEM) and supplemented with 10% fetal bovine serum (FBS). The pCXN2KS-Dsup was a gift from Prof. Kunieda Takekazu (Addgene plasmid #90019; http://n2t.net/addgene.90019 (10 January 2022); RRID: Addgene_90019). The empty vector (the same plasmid without Dsup) was kindly donated from Dr. Jlenia Brunetti (University of Siena). The expression construct and the empty plasmid were transfected into HEK293T cells using Lipofectamine^®^ 2000 Reagent (Life Technologies, Carlsbad, CA, USA), and stably transfected cells were selected using 700 µg/mL G418 (SERVA Electrophoresis GmbH, Heidelberg, Germany) treatment for three weeks. The evaluation of the Dsup transcript presence was assessed as described in previous work [8] and shown in Figure S4.

In order to evaluate the Dsup-induced resistance against radiation, both Dsup+ and Dsup− cells were plated at 100,000 cells/mL density in a 96-well plate. Before treatment, the complete medium was removed and replaced with 100 µL PBS and cells were exposed to UV-C for 15” (source 8W lamp–4 mJ/cm^2^, wavelength 260–280 nm, and source distance 5 cm). After treatment, cells were incubated in a complete medium for 24 h and then recovered [8].

### 4.2. Proteomic Analysis

Three independent cell cultures for basal and treatment conditions, in both cells transfected with Dsup (Dsup+) and an empty vector (Dsup−), were harvested via trypsinization and washed three times with phosphate buffer saline (PBS), with brief centrifugation (5 min at 1600× *g*) after each wash. Cell pellets were stored at −80 °C until proteomic analysis. Samples were dissolved in a lysis buffer (7 M Urea, 2 M Thiourea, 4% *w*/*v* 3-[(3-cholamidopropyl) dimethylammonia]-1-propanesulfonate hydrate (CHAPS), and 1% w/v dithioerythritol (DTE)) and protein concentration was determined using the Bradford assay. Samples were resolved using two-dimensional electrophoresis (2DE) according to Puglia M et al. [60]. Image analysis was carried out using Melanie™ Classic 9 (SIB Swiss Institute of Bioinformatics, Geneva, Swiss) and the percentage of relative volume (%V) of each spot was exported and used for statistical analysis. Statistically significant differences by ANOVA tests were determined via XLStat (Addinsoft, Paris, France) and processed according to the ratio value ≥ 2 and its corresponding %Vol means.

### 4.3. Protein Identification by MALDI-ToF Mass Spectrometry

Differential proteins found were identified via MALDI-ToF mass spectrometry using peptide mass fingerprinting (PMF). Differential spots were manually excized from MS-compatible silver-stained gels. Spots were destained first in a solution of 30 mM potassium ferricyanide and 100 mM sodium sulphate anhydrous, and then later in 200 mM ammonium bicarbonate. Then, they were dehydrated in 100% acetonitrile (ACN). Protein spots were rehydrated and digested overnight at 37 °C in a trypsin solution. Digested proteins were then placed on a MALDI target, dried, and covered with a matrix solution of 5 mg/mL α-cyano-4-hydroxycinnamic acid (CHCA) in 50% *v*/*v* ACN and 0.5% *v*/*v* trifluoroacetic acid (TFA). The MS analysis was carried out using the UltrafleXtreme™ MALDI-ToF/ToF mass spectrometer (Bruker Daltoniks, Bremen, Germany), equipped with a 200 Hz smartbeam™ I laser in the positive reflector mode with the following parameters: 80 ns of delay; ion source 1: 25 kV; ion source 2: 21.75 kV; lens voltage: 9.50 kV; reflector voltage: 26.30 kV; and reflector 2 voltage: 14.00 kV. The applied laser wavelength and frequency were 353 nm and 100 Hz, respectively, and the percentage was set to 50%. Final mass spectra were produced by averaging 1500 laser shots targeting five different positions within the spot. MS spectra were acquired and processed via the FleXanalysis software version 3.0 (Bruker) using peptides arising from trypsin autoproteolysis as the internal standard for calibration. The resulting mass lists were filtered for common contaminants such as matrix-related ions, trypsin autolysis, and keratin peaks. Protein identification was carried out by utilizing the peptide mass fingerprinting search using MASCOT (Matrix Science Ltd., London, UK, http://www.matrixscience.com; accessed on 2 May 2022); setting up the following parameters: Homo sapiens as taxonomy, SwissProt as database, 20 ppm as mass tolerance, one admissible missed cleavage site, and carbamidomethylation (iodoacetamide alkylation) of cysteine as fixed modification and oxidation of methionine as a variable modification. Only protein identifications with a *p*-value < 0.04 (referred to as “expect”, as reported in Tables S1 and S2), a minimun of four matched peptides and a minimum MASCOT score of 55 were considered. The mass spectrometry proteomics data have been deposited to the ProteomeXchange Consortium via the PRIDE [61] partner repository with the dataset identifier, PXD037439.

### 4.4. Telomere Length

Relative telomere length (RTL) was quantified via qPCR on live cells. Briefly, culture medium was removed from cell cultures and the adhering, living cells were washed with PBS to remove possible debris and death cells. Once PBS was removed, adhering cells were harvested via trypsinization and DNA was extracted using a QIAamp DNA mini kit (Qiagen, Hilden, Germany) following kit instructions. RTL was quantified on 100 ng/µL of DNA by determining the relative ratio of the telomere (T) repeat copy number to a single copy gene (S) copy number (T/S ratio). This ratio is proportional to the average telomere length [62]. The encoding acidic ribosomal phosphoprotein P0, 36B4, was used as the single copy gene. The primers were as follows and were used at a 300 nM final concentration in a reaction mix containing 12.5 µL SYBER GREEN PCR Master Mix for a final volume of 25 µL: telomere sense, 5′-GGTTTTTTGAGGGTGAGGGTGAGGGTGAGGGTGAGGGT-3′; telomere antisense, 5′-TCCCCGACTATCCCTATCCCTATCCCTATCCCTATCCCTA-3′; 36B4 sense, 5′-CCCATTCTATCACAACGGTACAA-3′; and 36B4 antisense, 5′-CAGCAAGTGGGAAGGTGTAATCC-3′. The thermal cycling profile for the telomere amplification was 95 °C for 10 min, followed by 30 cycles of 95 °C for 15 s and 54 °C for 1 min. For the 36B4 amplification, it was 95 °C for 10 min, followed by 40 cycles of 95 °C for 15 s and 60 °C for 1 min. To exclude the presence of nonspecific binding between SYBER GREEN and primers, a melting curve was added at the end of all PCR-amplification reactions.

### 4.5. Western Blot

Twenty-five micrograms of each protein sample was suspended in 20 µL of LAEMMLI (Tris HCl pH 6.8 62.5 mM, 20% *v*/*v* glycerol, 2% *w*/*v* SDS, 5% *v*/*v* β-mercaptoethanol, and 0.032% *w*/*v* bromophenol blue), heated at 95 °C for 7 min, and loaded in 12% of polyacrylamide gels. Monodimensional electrophoresis was carried out using the Mini-PROTEAN electrophoresis system (Bio-Rad, Heracles, CA, USA). After SDS-PAGE, gels were equilibrated for 1 h in transfer buffer and then transferred to nitrocellulose membranes (Cytiva, Washington, DC, USA). After transfer, membranes were stained using Red Ponceau (0.2% *w*/*v* Ponceau S in 3% acetic acid) to assess the correct and equal protein loading as well as the correct protein transfer. Membranes were tested with different primary antibodies, such as anti-HSP90 (mouse monoclonal, 610418, BD Transduction Laboratories, Hoboken, NJ, USA, dilution 1:1000), anti-ERP29 (rabbit polyclonal, ALX-210-404-R100, Enzo Life Sciences, Farmingdale, NY, USA, dilution 1:2500), anti-ENOA (mouse monoclonal, sc-100812 Santa Cruz biotechnology, Dallas, TX, USA, dilution 1:200), anti c-Myc (rabbit polyclonal, ab69987, Abcam, Cambridge, UK, dilution 1:1000), anti-PPIA (rabbit polyclonal, LS-C36063, LifeSpan Biosciences, Seattle, WA, USA, dilution 1:1000), and anti-ARP3 (rabbit polyclonal, sc-15390, Santa Cruz Biotechnology, Dallas, TX, USA, dilution 1:200). Membranes were incubated with different secondary antibodies according to specific antibody composition, such as peroxidase-conjugated goat anti-rabbit at 1:7000 dilution and peroxidase-conjugated goat anti-mouse at 1:3000 dilutions.

Considering that, via proteomic analysis, only one proteoform of β-actin in Dsup−/+ cells at basal condition varies in abundance, Western blot normalization against total β-actin in full was performed (anti-β-actin mouse monoclonal, A5441, Sigma, New York, NY, USA, dilution: 1:40,000). The ECL chemiluminescence detection system (Cytiva) was utilized and specific immunoreactive proteins and images were analyzed with ImageJ (1.51p, NIH, Chicago, IL, USA) to obtain band intensity. Statistical analysis was performed via XLSTAT version 2021.2.2 (Addinsoft).

### 4.6. Statistical and Enrichment Analyses

A multivariate analysis using principal component analysis (PCA) via Pearson’s correlation and a heatmap analysis using the Euclidean distance were performed for basal and UV-C treatment, comparing Dsup− and Dsup+ cells, considering the %V of statistically significant differential spots via XLStat (Addinsoft). Enrichment analysis was performed by submitting the UniProt accession numbers (AN) of the identified proteins into the MetaCore software (https://portal.genego.com, accessed on 23 May 2022) (Clarivate analytics, Boston, MA, USA). For Dsup+ vs. Dsup− comparison, the protein network analysis was then carried out using the “shortest path” algorithm that builds a hypothetical network connecting two experimental proteins directly or indirectly using one or more MetaCore database proteins. This algorithm allows building a network including only closely related proteins and introducing a maximum of one non-experimental protein prioritized according to their statistical significance (*p* ≤ 0.001). Networks were visualized graphically as nodes (proteins) and edges (links between proteins) and relevant biological processes were then prioritized according to their statistical significance (*p* ≤ 0.001 and FDR) to report the specific proteins involved (the legend is fully explained in Figure S5). The GO biological process enrichment analysis was also performed. Moreover, the Map folder tool was used to highlight well-known molecular pathway regulation processes including our differential proteins, as well as the enrichment via process networks and pathway maps. For telomere length, statistical analysis was performed with StatView for Windows, version 5.00.1 (SAS Institute, Cary, NC, USA). A one-way ANOVA with Fisher’s correction was used to determine the RTL difference as a continuous variable. Survival data were analyzed using a paired test for non-parametric data (Wilcoxon signed-rank test). For all comparisons, a *p* value of < 0.05 was considered significant.

## 5. Conclusions

Our study shows that in transfected-human cells, Dsup mediates cell protection against the damaging effects of UV-C by activating more efficient mechanisms of DNA-damage repair, mRNA stability, telomere elongation and maintenance, unfolded protein response, and cytoplasmic stress granule response together with a metabolic modulation. These promising data are, however, just a little part of the still unknown cellular pathways involved in Dsup-mediated protection from external stimulus. Therefore, investigations must proceed forward.

## 6. Limitations

The top-down approach via 2DE was used to perform proteomic analyses in order to more comprehensively evaluate the entire proteome of samples of interest, including proteolytic products and PTM proteoforms. However, this technique has some limitations: it is a semi-quantitative method relying on the relative quantitation of each protein species via image analysis, although normalization of the spots’ relative volumes is performed by considering the % of relative volume. Moreover, two-dimensional focusing might sometimes result in the streaking of high-abundance protein species as well as an overlapping of extremely closed proteoforms, interfering with proper protein identification and discrimination. These experimental events have been overcome by performing protein identification of the same spot from multiple preparative gels in order to evaluate correct identification from potential protein mixtures arising from the same spot. In addition, due to the chosen experimental approach, MALDI-ToF mass spectrometry has been used for protein identification of protein species coming from the 2DE gel spots as it allows rapid, reliable, and sensitive identification of proteins. As identification relies on the matching of a list of peptide masses against protein sequence databases, we applied more stringent inclusion criteria as a *p*-value < 0.04, resulting in a minimum of four matched peptides, a peptide tolerance mass of 20 ppm, and a minimum MASCOT score of 55. Given these limitations, this approach has been applied to these preliminary analyses and the obtained proteomic data results are worthy of further analyses.

## Data Availability

All mass-spectrometry-proteomics data have been deposited into the ProteomeXchange Consortium via the PRIDE partner repository with the dataset identifier, PXD037439. All other relevant data are within the paper and its Supporting Information files. Requests for further information about resources, reagents, and data availability should be directed to the corresponding author.

## Supplementary Materials

The supporting information can be downloaded at: https://www.mdpi.com/article/10.3390/ijms241411463/s1.
