# Supplementary material for: Proteomics Reveals How the Tardigrade Damage Suppressor Protein Teaches Transfected Human Cells to Survive UV-C Stress
Source: Int J Mol Sci. 2023 Jul 14;24(14):11463. doi: 10.3390/ijms241411463 (PMC10380570; doi:10.3390/ijms241411463)
Supplement: Supplementary file 1 [file ijms-24-11463-s001.zip › Shaba et al supplementary figures.pdf]

Proteomics reveals how the tardigrade damage suppressor protein teaches transfected human cells how to struggle with UV-C stress

Enxhi Shaba[1*], Claudia Landi[1*§], Carlotta Marzocchi[2], Lorenza Vantaggiato[1], Luca Bini[1], Claudia Ricci[2^] and Silvia Cantara[2^]

[1]Functional Proteomics Lab, Department of Life Sciences, University of Siena, 53100 Siena, Italy.
[2]Department of Medical, Surgical and Neurological Sciences, University of Siena, 53100 Siena, Italy.
* co-first authors
^ co-last authors

Supporting Information: This article contains supporting information, as listed below:

Figure S1: Representative 2DE electropherograms of Dsup− vs Dsup+ HEK293T cells at basal condition;

Figure S2: Survival assay of HEK293T cells control (Dsup-) and Dsup-transfected (Dsup+) after 15” of UV-C exposure recovered after 24h. The dotted line represents the basal condition (100%)

Figure S3: Representative 2DE electropherograms of Dsup− vs Dsup+ HEK293T cells exposed to 15 seconds of UV-C radiation and recovered after 24h;

Figure S4: Dsup assessment. Dsup expression in control (Dsup−) and transfected (Dsup+) HEK293T cells was performed by endpoint PCR and visualized in 2% agarose gel by ethidium bromide;

Figure S5: MetaCore legend;

Table S1: Proteomic results of the Dsup− vs Dsup+ HEK293T cells analysis at basal condition

Table S2: Proteomic result of the Dsup− vs Dsup+ HEK293T cells analysis exposed to UV-C

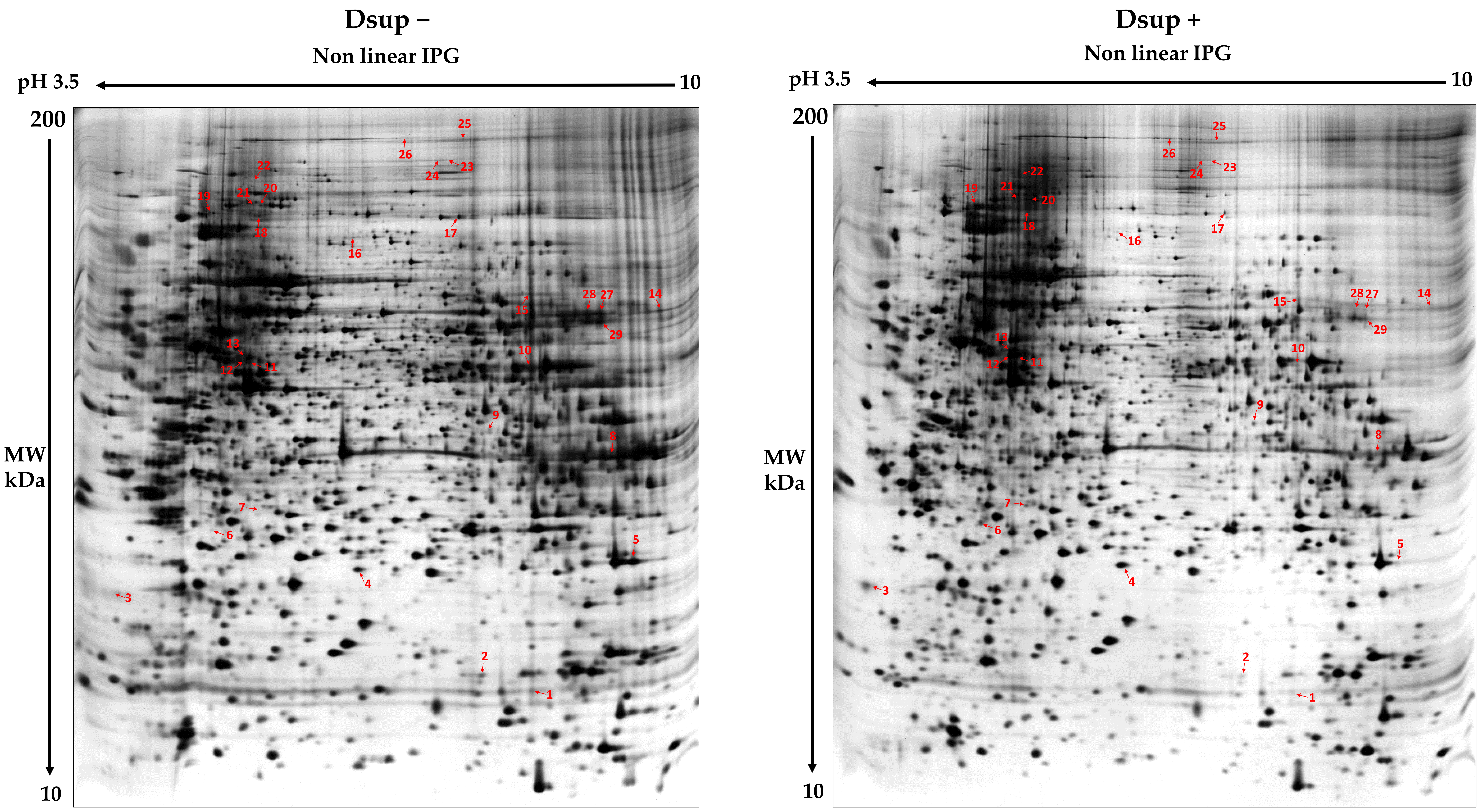

Figure S1: Representative 2DE electropherograms of Dsup– vs Dsup+ HEK293T cells at basal condition. The differentially abundant spots between the two considered conditions are reported in red. Spot numbers correspond to those showed in Table S1**.**

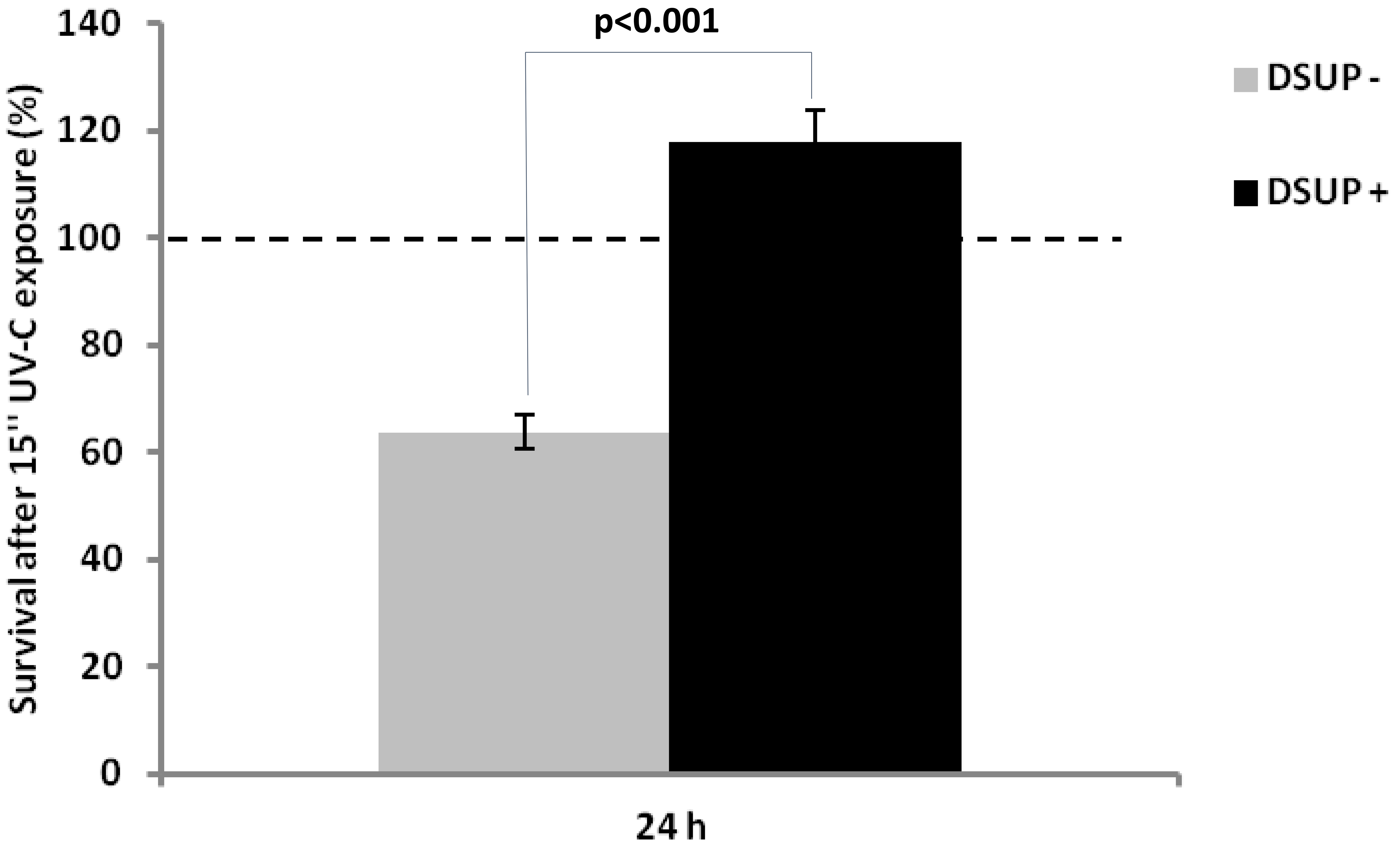

Figure S2: Survival assay of HEK293T cells control (Dsup-) and Dsup-transfected (Dsup+) after 15” of UV-C exposure recovered after 24h. The dotted line represents the basal condition (100%)

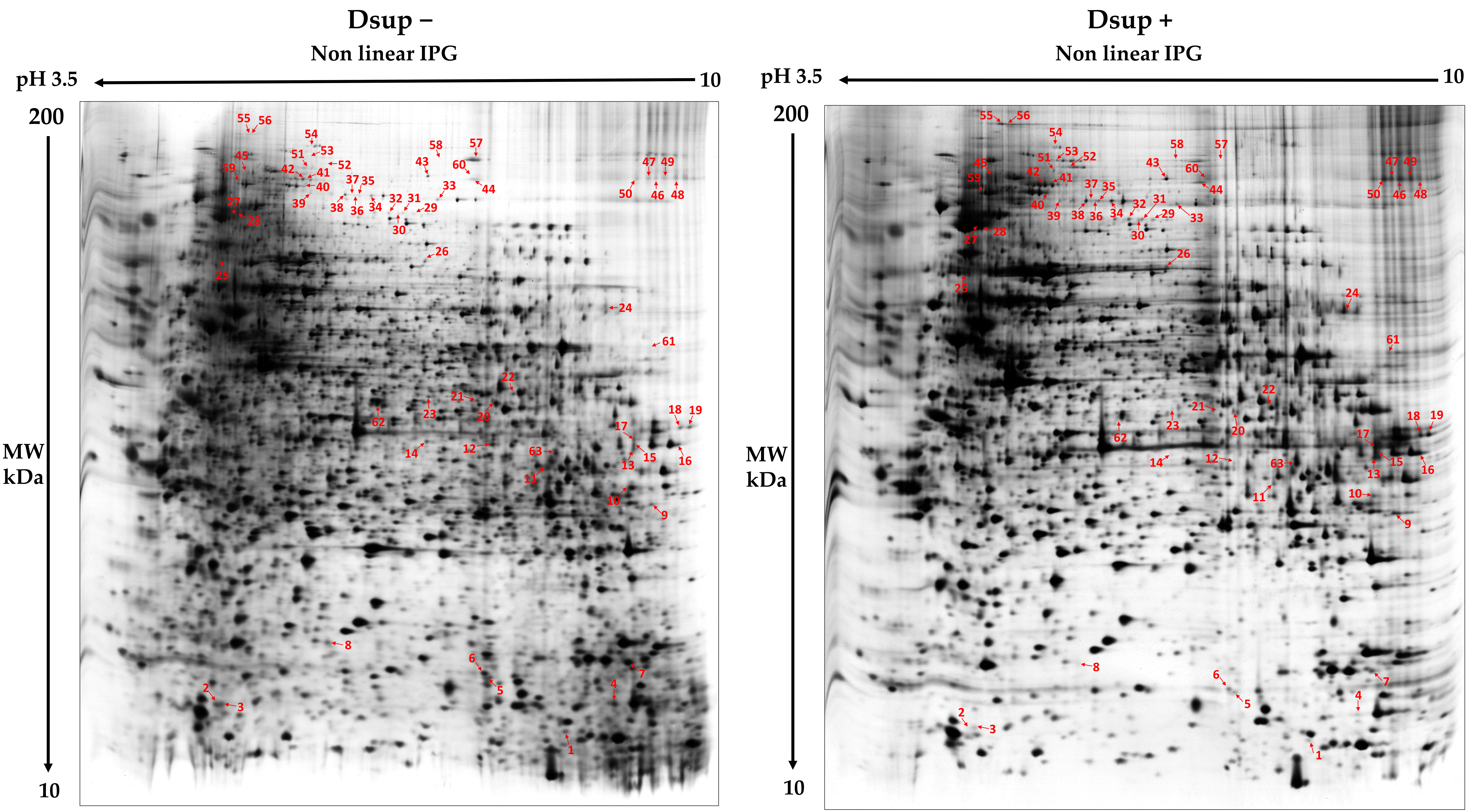

Figure S3: Representative 2DE electropherograms of Dsup– vs Dsup+ HEK293T cells exposed to 15 seconds of UV-C radiation and recovered after 24h. The differentially abundant spots between the two considered conditions are reported in red. Spot numbers correspond to that in Table S2.

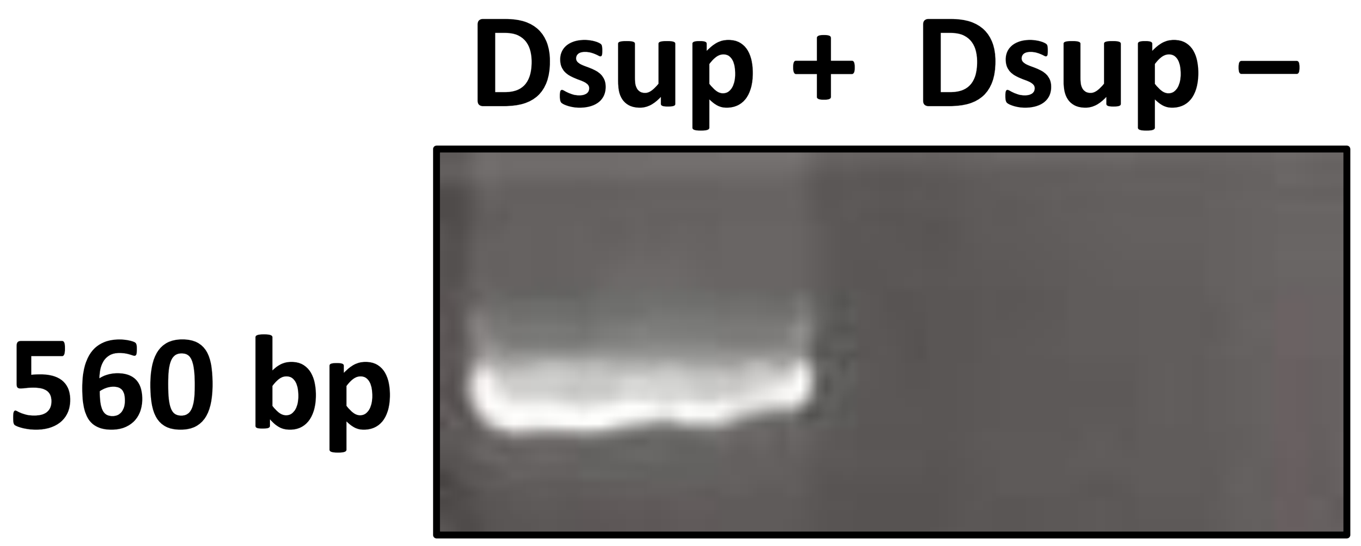

Figure S4. Dsup expression assessment. Dsup expression in control (Dsup−) and transfected (Dsup+) HEK293T cells was performed by endpoint PCR and visualized in 2% agarose gel by ethidium bromide.

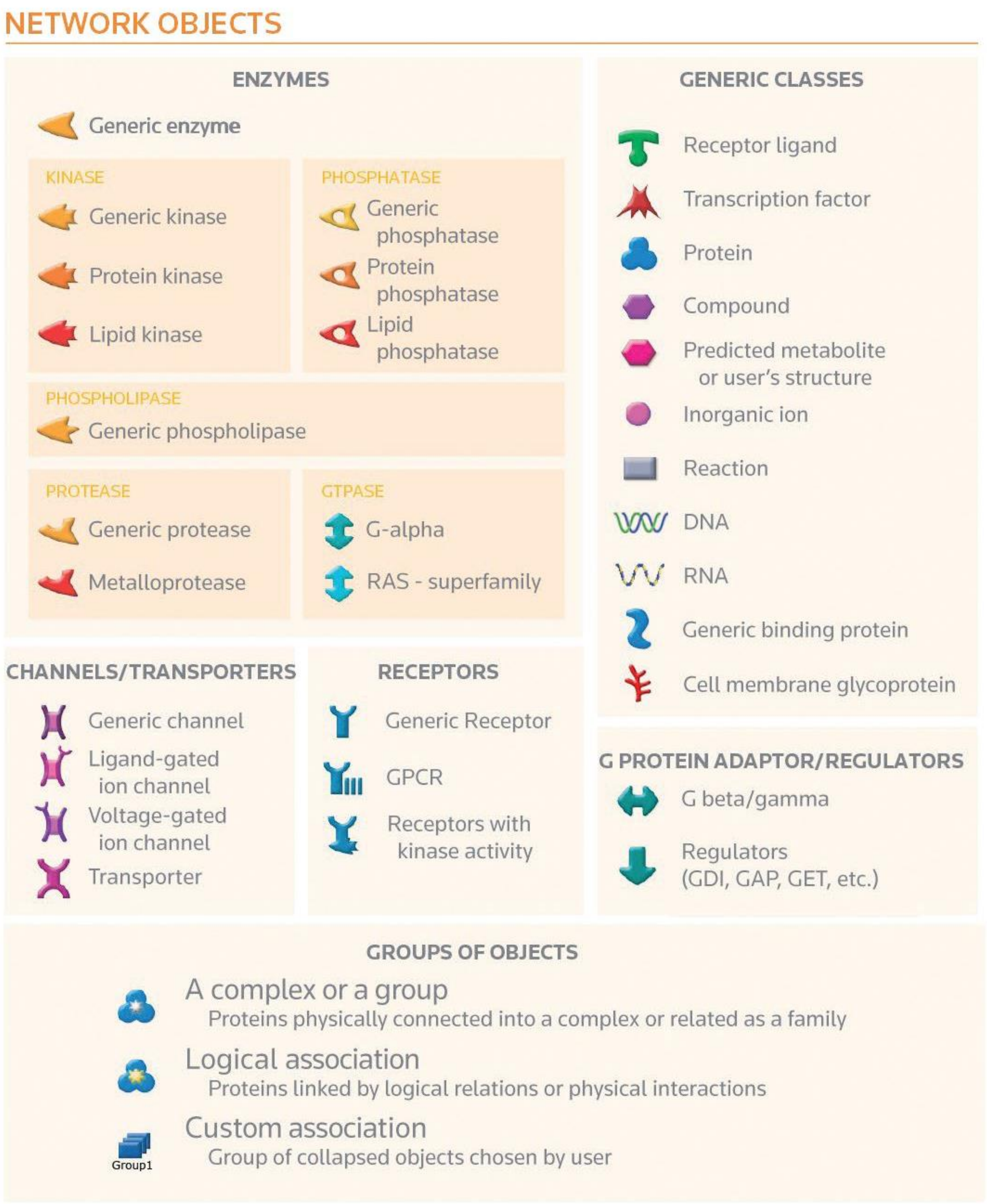

Figure S5: Metacore legend
