# Supplementary material for: Proteomics Reveals How the Tardigrade Damage Suppressor Protein Teaches Transfected Human Cells to Survive UV-C Stress
Source: Int J Mol Sci. 2023 Jul 14;24(14):11463. doi: 10.3390/ijms241411463 (PMC10380570; doi:10.3390/ijms241411463)
Supplement: Supplementary file 1 [file ijms-24-11463-s001.zip › Shaba et al. supplementary tables.pdf]

Table S1. Proteomic data and identifications of differential spots in Dsup–/+ HEK293T cells at basal condition

Table reports the spot number corresponding to that in Figure S3, protein name when the protein was identified by MALDI-ToF MS, UniProt Entry name and accession number (AC), the ANOVA Test and the mean of the %V of the specific spot in Dsup− and Dsup+ cells, the fold change of the %V means of Dsup− vs Dsup+ and viceversa, pI and MW. Last part of the table is dedicated to Mascot Search Results such as Score and Expect, Matched Peptides and sequence Coverage (%).

Spot n°	Protein name	Entry name	AC	Anova test			Ratio		pI - MW	Mascot Search Results		
				Anova (p)	(Basal) Dsup−	(Basal) Dsup+	(Basal) Dsup−/+	(Basal) Dsup+/−		Score Expect	Matched peptides	Coverage (%)
**1**				0.003	0.043355	0.016045	**2.702161872**	0.370074054				
**2**				0.011	0.019463	0.007949	**2.448426838**	0.408425518				
**3**	**Prostaglandin E synthase 3**	**TEBP_HUMAN**	**Q15185**	0.040	0.025233	0.050755	0.497158563	**2.011430708**	4.35 - 18971	**128** $3.2E^{-09}$	8/12	45
**4**	**Heat shock 70 kDa protein 1A** N-term fragment	**HS71A_HUMAN**	**P0DMV8**	0.009	0.074455	0.165706	0.449321792	**2.225576452**	5.48 - 70294	**224**<br>**169** $2.6E^{-13}$	17/30	26
	**Heat shock cognate 71 kDa protein** N-term fragment	**HSP7C_HUMAN**	**P11142**						5.37 - 71082	**89** $2.3E^{-05}$	12/30	21
**5**	**Peroxiredoxin-1**	**PRDX1_HUMAN**	**Q06830**	0.018	0.161855	0.040361	**4.010171485**	0.249365895	8.27 - 22324	**230** $2E^{-19}$	13/15	62
**6**	**Heat shock 70 kDa protein 1A** C-term fragment	**HS71A_HUMAN**	**P0DMV8**	0.007	0.006086	0.038048	0.159949258	**6.251982753**	5.48 - 70294	**111** $1.6E^{-07}$	10/14	16
**7**	**Pyruvate kinase PKM** N-term fragment	**KPYM_HUMAN**	**P14618**	0.010	0.002012	0.0199	0.101115909	**9.889640595**	7.96 - 58470	**152** $1.3E^{-11}$	12/20	26
**8**	**Heterogeneous nuclear ribonucleoproteins A2/B1**	**ROA2_HUMAN**	**P22626**	0.036	0.07028	0.030466	**2.306830143**	0.433495289	8-97 - 37464	**173** $1E^{-13}$	12/15	42
**9**	**Pyruvate kinase PKM** N-term fragment	**KPYM_HUMAN**	**P14618**	0.046	0.004739	0.030441	0.155674606	**6.423655244**	7.96 - 58470	**181** $1.6E^{-14}$	14/17	27

**10**	**Alpha-enolase**	**ENOA_HUMAN**	**P06733**	0.037	0.018212	0.008153	**2.23389549**	0.447648516	7.01 - 47481	**122** $1.3E^{-08}$	8/10	16
**11**	**Actin. cytoplasmic 1 / Actin. cytoplasmic 2**	**ACTB_HUMAN / ACTG_HUMAN**	**P60709 / P63261**	0.037	0.046185	0.10382	0.444861478	**2.247890748**	5.29 - 42052<br>5.31 - 42108	**197** $4.1E^{-16}$	18/30	45
**12**	**Eukaryotic initiation factor 4A-II**	**IF4A2_HUMAN**	**Q14240**	0.012	0.043265	0.132976	0.325356519	**3.073551449**	5.33 - 46601	**93** $9.3E^{-06}$	10/28	26
**13**	**Eukaryotic initiation factor 4A-II**	**IF4A2_HUMAN**	**Q14240**	0.005	0.020374	0.046276	0.440278618	**2.27128904**	5.33 - 46601	**75** $6.4E^{-04}$	9/32	25
**14**				0.004	0.051314	0.025473	**2.014449267**	0.496413594				
**15**	**Asparagine synthetase [glutamine-hydrolyzing]**	**ASNS_HUMAN**	**P08243**	0.030	0.058045	0.027783	**2.089232774**	0.478644607	6.39 - 64899	**139** $2.6E^{-10}$	9/10	14
**16**				0.020	0.005422	0.002635	**2.057749068**	0.485967903				
**17**				0.049	0.055061	0.02651	**2.076969696**	0.481470674				
**18**	**Striatin-4**	**STRN4_HUMAN**	**Q9NRL3**	0.047	0.008072	0.020211	0.399375914	**2.503906631**	5.21 - 81287	**103** $1E^{-06}$	8/11	13
**19**	**Heat shock protein HSP 90-beta**	**HS90B_HUMAN**	**P08238**	0.020	0.013532	0.038488	0.351600345	**2.844138278**	4.97 - 83554	**168** / **106** $5.1E^{-07}$	14/36	21
	**Cleavage and polyadenylation specificity factor subunit 2**	**CPSF2_HUMAN**	**Q9P2I0**						4.98 - 89286	**84** $7.7E^{-05}$	15/36	16
**20**	**DNA replication licensing factor MCM6**	**MCM6_HUMAN**	**Q14566**	0.049	0.014905	0.033412	0.446097602	**2.241661905**	5.29 - 93801	**163** $1E^{-12}$	16/25	19
**21**	**DNA replication licensing factor MCM6**	**MCM6_HUMAN**	**Q14566**	0.043	0.020144	0.041181	0.489161616	**2.044314124**	5.29 - 93801	**146** $5.1E^{-11}$	13/17	15
**22**				0.027	0.006606	0.020521	0.321895365	**3.106599559**				
**23**				0.013	0.000986	0.002336	0.421981066	**2.369774571**				
**24**				0.012	0.001525	0.004027	0.378789183	**2.639990909**				
**25**				0.045	0.002205	0.004461	0.494212097	**2.023422748**				

**26**				0.035	0.002161	0.00501	0.431435301	**2.31784464**				
**27**	**Tyrosine--tRNA ligase. cytoplasmic**	**SYYC_HUMAN**	**P54577**	0.027	0.097784	0.036367	**2.688779653**	0.371915935	6.61 - 59448	**118**<br>$3.2E^{-08}$	9/13	25
**28**	**Tyrosine--tRNA ligase. cytoplasmic**	**SYYC_HUMAN**	**P54577**	0.027	0.183837	0.081087	**2.267169064**	0.441078707	6.61 - 59448	**119**<br>$2.6E^{-08}$	10/15	21
**29**	**T-complex protein 1 subunit delta**	**TCPD_HUMAN**	**P50991**	0.013	0.174077	0.066644	**2.612048545**	0.382841277	7.96 - 58401	**161**<br>$1.6E^{-12}$	13/16	27

Table S2: Proteomic data and identifications of differential spots in Dsup–/+ HEK293T cells at 24h recovery after 15 seconds of UV-C radiation

Table reports the spot number corresponding to that in Figure S4, protein name when the protein was identified by MALDI-ToF MS, UniProt Entry name and accession number (AC), the ANOVA Test and the mean of the %V of the specific spot in Dsup− and Dsup+ cells, the fold change of the %V means of Dsup− vs Dsup+ and viceversa, pI and MW. Last part of the table is dedicated to Mascot Search Results such as Score and Expect, Matched Peptides and sequence Coverage (%).

<table>
<tr><th rowspan="2">Spot n°</th><th rowspan="2">Protein name</th><th rowspan="2">Entry name</th><th rowspan="2">AC</th><th colspan="3">Anova test</th><th colspan="2">Ratio</th><th rowspan="2">pI - MW</th><th colspan="4">Mascot Search Results</th></tr>
<tr><th>Anova (p)</th><th>(UV) Dsup−</th><th>(UV) Dsup+</th><th>(UV) Dsup −/+</th><th>(UV) Dsup +/−</th><th colspan="2">Score Expect</th><th>Matched peptides</th><th>Coverage (%)</th></tr>
<tr><td>1</td><td colspan="3"></td><td>0.030</td><td>0.021045</td><td>0.0093442</td><td>**2.252196461**</td><td>0.444010999</td><td colspan="5"></td></tr>
<tr><td rowspan="2">2</td><td>**Regulator complex protein LAMTOR2**</td><td>**LTOR2_HUMAN**</td><td>**Q9Y2Q5**</td><td rowspan="2">0.026</td><td rowspan="2">0.0190797</td><td rowspan="2">0.0040741</td><td rowspan="2">**4.683227283**</td><td rowspan="2">0.213527967</td><td>5.30 - 13613</td><td rowspan="2">**103**</td><td>**78** $2.9E^{-04}$</td><td>5/14</td><td>38</td></tr>
<tr><td>**Replication protein A 14 kDa subunit**</td><td>**RFA3_HUMAN**</td><td>**P35244**</td><td>4.96 - 13674</td><td>**63** $9.5E^{-03}$</td><td>4/14</td><td>54</td></tr>
<tr><td>3</td><td>**Thioredoxin**</td><td>**THIO_HUMAN**</td><td>**P10599**</td><td>0.013</td><td>0.0270251</td><td>0.0090355</td><td>**2.99097785**</td><td>0.334338818</td><td>4.82 - 12015</td><td colspan="2">**69** $2.8E^{-03}$</td><td>5/7</td><td>32</td></tr>
<tr><td>4</td><td colspan="3"></td><td>0.050</td><td>0.0481777</td><td>0.011223</td><td>**4.292765278**</td><td>0.232950077</td><td colspan="5"></td></tr>
<tr><td>5</td><td colspan="3"></td><td>0.005</td><td>0.0467749</td><td>0.0196331</td><td>**2.382453412**</td><td>0.419735385</td><td colspan="5"></td></tr>
<tr><td>6</td><td colspan="3"></td><td>0.028</td><td>0.0379288</td><td>0.0156038</td><td>**2.430736037**</td><td>0.411398023</td><td colspan="5"></td></tr>
<tr><td>7</td><td>**Peptidyl-prolyl cis-trans isomerase A**</td><td>**PPIA_HUMAN**</td><td>**P62937**</td><td>0.015</td><td>0.0833672</td><td>0.0167316</td><td>**4.982629633**</td><td>0.200697237</td><td>7.68-18229</td><td colspan="2">**182** $1.3E^{-14}$</td><td>13/26</td><td>58</td></tr>
<tr><td>8</td><td>**Actin-related protein 2/3 complex subunit 5**</td><td>**ARPC5_HUMAN**</td><td>**O15511**</td><td>0.030</td><td>0.0273185</td><td>0.0094444</td><td>**2.892558075**</td><td>0.345714753</td><td>5.47-16367</td><td colspan="2">**146** $5.1E^{-11}$</td><td>11/17</td><td>52</td></tr>
<tr><td>9</td><td>**Peroxiredoxin-1**</td><td>**PRDX1_HUMAN**</td><td>**Q06830**</td><td>0.022</td><td>0.0184992</td><td>0.0615035</td><td>0.300783424</td><td>**3.324651292**</td><td>8.27- 22324</td><td colspan="2">**100** $2E^{-06}$</td><td>7/16</td><td>35</td></tr>
<tr><td>10</td><td colspan="3"></td><td>0.042</td><td>0.0711136</td><td>0.0263909</td><td>**2.694627939**</td><td>0.371108748</td><td colspan="5"></td></tr>
<tr><td>11</td><td>**S-**</td><td>**ESTD_HUMAN**</td><td>**P10768**</td><td>0.037</td><td>0.0474349</td><td>0.0213141</td><td>**2.225519297**</td><td>0.449333331</td><td>6.54-31956</td><td colspan="2">**158**</td><td>11/17</td><td>50</td></tr>
</table>

	**formylglutathione hydrolase**									3.2E^{-12}		
**12**	**Phosphatidylinositol transfer protein beta isoform**	**PIPNB_HUMAN**	**P48739**	0.010	0.0342307	0.0064848	**5.278594214**	0.189444378	6.41-31805	**356** 5.1E^{-32}	23/26	67
**13**	**Heterogeneous nuclear ribonucleoproteins A2/B1**	**ROA2_HUMAN**	**P22626**	0.010	0.0270482	0.0973908	0.277728245	**3.600642064**	8.97-37464	**143** 1E^{-10}	10/15	38
**14**				0.049	0.0118442	0.0021141	**5.602421492**	0.178494246				
**15**	**Heterogeneous nuclear ribonucleoproteins A2/B1**	**ROA2_HUMAN**	**P22626**	0.001	0.0135462	0.0539949	0.250879095	**3.985983769**	8.97-37464	**213** 1E^{-17}	14/17	42
**16**	**Heterogeneous nuclear ribonucleoproteins A2/B1**	**ROA2_HUMAN**	**P22626**	0.008	0.0281454	0.0620498	0.453592945	**2.204619825**	8.97-37464	**114** 8.1E^{-08}	8/13	35
**17**	**Heterogeneous nuclear ribonucleoproteins A2/B1**	**ROA2_HUMAN**	**P22626**	0.042	0.0395889	0.1261191	0.313901255	**3.1857152**	8.97-37464	**186** 5.1E^{-15}	13/18	42
**18**	**Heterogeneous nuclear ribonucleoproteins A2/B1**	**ROA2_HUMAN**	**P22626**	<0.0001	0.0243909	0.064495	0.378182093	**2.644228851**	8.97-37464	**344** 8.1E^{-31}	23/25	53
**19**	**Heterogeneous nuclear ribonucleoproteins A2/B1**	**ROA2_HUMAN**	**P22626**	<0.0001	0.0170888	0.0497053	0.343801701	**2.908653443**	8.97-37464	**167** 4.1E^{-13}	11/14	42
**20**	**Poly(rC)-binding protein 1**	**PCBP1_HUMAN**	**Q15365**	0.035	0.0688577	0.0337199	**2.042048324**	0.489704376	6.66-37987	**137** 4.1E^{-10}	10/15	41
**21**				0.016	0.0061338	0.0013465	**4.555468387**	0.219516395				
**22**	**Mitogen-activated protein kinase**	**MK01_HUMAN**	**P28482**	0.019	0.0164557	0.0342318	0.480713334	**2.080241862**	6.50 - 41762	**175** | **126** 5.1e^{-09}	10/20	24

	**1**											
	**Sialic acid synthase**	**SIAS_HUMAN**	**Q9NR45**						6.29 - 40738	**90** $2.2E^{-05}$	8/20	23
**23**	**Translation initiation factor eIF-2B subunit beta**	**EI2BB_HUMAN**	**P49770**	0.047	0.0113539	0.0031515	**3.602685479**	0.277570719	5.77-39193	**216** $5.1E^{-18}$	17/24	45
**24**	**D-3-phosphoglycerate dehydrogenase**	**SERA_HUMAN**	**O43175**	0.003	0.022067	0.07731	0.285434953	**3.50342518**	6.29-57356	**160** $2E^{-12}$	14/24	31
**25**	**Heat shock 70 kDa protein 1A**	**HS71A_HUMAN**	**P0DMV8**	0.037	0.03986	0.0879722	0.453097683	**2.207029604**	5.48- 70294	**275** $6.4E^{-24}$	21/29	43
	**Heat shock 70 kDa protein 1B**	**HS71B_HUMAN**	**P0DMV9**						5.48- 70294	**275** $6.4E^{-24}$	21/29	43
**26**	**Heat shock 70 kDa protein 1A**	**HS71A_HUMAN**	**P0DMV8**	0.047	0.0059819	0.0130175	0.459529213	**2.176140214**	5.48- 70294	**161** $1.6E^{-12}$	12/15	23
	**Heat shock 70 kDa protein 1B**	**HS71B_HUMAN**	**P0DMV9**						5.48- 70294	**161** $1.6E^{-12}$	12/15	23
**27**	**Heat shock protein HSP 90-beta**	**HS90B_HUMAN**	**P08238**	0.017	0.0833278	0.1669537	0.499107138	**2.003577839**	4.97-83554	**169** $2.6E^{-13}$	20/35	29
**28**	**Heat shock protein HSP 90-beta**	**HS90B_HUMAN**	**P08238**	0.032	0.040419	0.0918989	0.439820484	**2.273654904**	4.97-83554	**169** $2.6E^{-13}$	20/35	29
**29**	**Cold shock domain-containing protein E1**	**CSDE1_HUMAN**	**O75534**	0.048	0.003588	0.0085877	0.417811	**2.393426693**	5.88-89684	**117** $4.1E^{-08}$	11/17	17
**30**	**Cold shock domain-containing protein E1**	**CSDE1_HUMAN**	**O75534**	0.003	0.0022853	0.0064755	0.352919859	**2.833504472**	5.88-89684	**236** $5.1E^{-20}$	21/28	28
**31**	**Cold shock domain-**	**CSDE1_HUMAN**	**O75534**	0.047	0.0035469	0.0075604	0.469145453	**2.131535099**	5.88-89684	**101** $1.6E^{-06}$	9/13	13

	**containing protein E1**											
**32**	**Cold shock domain-containing protein E1**	**CSDE1_HUMAN**	**O75534**	0.048	0.0046699	0.0097517	0.478884216	**2.088187429**	5.88-89684	**74** 7.9E^{-04}	8/18	14
**33**	**Elongation factor 2**	**EF2_HUMAN**	**P13639**	0.032	0.0081677	0.0196089	0.416530617	**2.400783901**	6.41-96246	**178** 3.2E^{-14}	16/20	21
**34**	**Neutral alpha-glucosidase AB**	**GANAB_HUMAN**	**Q14697**	0.028	0.0068976	0.0216881	0.318036493	**3.144293254**	5.74-107263	**316** 5.1E^{-28}	29/34	32
**35**	**Heat shock 70 kDa protein 4L**	**HS74L_HUMAN**	**O95757**	0.016	0.0040901	0.0144419	0.283208476	**3.530967761**	5.63 - 95479	**61** 1.5E^{-02}	5/6	8
**36**	**Heat shock 70 kDa protein 4L**	**HS74L_HUMAN**	**O95757**	0.028	0.0016363	0.0072614	0.225338917	**4.437759864**	5.63-95479	**124** 8.1E^{-09}	13/20	16
**37**	**Heat shock 70 kDa protein 4L**	**HS74L_HUMAN**	**O95757**	0.021	0.0012532	0.0049527	0.253033774	**3.952041592**	5.63-95479	**124** 8.1E^{-09}	13/20	16
**38**	**Neutral alpha-glucosidase AB**	**GANAB_HUMAN**	**Q14697**	0.021	0.0079115	0.0167872	0.471280358	**2.121879224**	5.74-107263	**97** 2.9E^{-06}	10/17	12
**39**	**Ubiquitin-like modifier-activating enzyme 1**	**UBA1_HUMAN**	**P22314**	0.015	0.0010479	0.0048447	0.216293867	**4.623339591**	5.49 - 118858	**57** 3.9E^{-02}	4/4	6
**40**	**Ubiquitin-like modifier-activating enzyme 1**	**UBA1_HUMAN**	**P22314**	0.019	0.0099294	0.0352576	0.281624783	**3.550823865**	5.49- 118858	**307** 4.1E^{-27}	25/30	35
**41**	**Ubiquitin-like modifier-activating enzyme 1**	**UBA1_HUMAN**	**P22314**	0.008	0.0075397	0.033307	0.226370933	**4.417528287**	5.49- 118858	**165** 6.4E^{-13}	17/29	24
**42**	**Ubiquitin-like modifier-activating enzyme 1**	**UBA1_HUMAN**	**P22314**	0.001	0.0120293	0.0314904	0.381999189	**2.617806604**	5.49- 118858	**303** 1E^{-26}	25/31	36
**43**	**Transcription intermediary factor 1-beta**	**TIF1B_HUMAN**	**Q13263**	0.018	0.0103278	0.0218028	0.473691565	**2.111078334**	5.52-90261	**77** 3.7E^{-04}	7/11	10
**44**				0.010	0.0121098	0.0732296	0.165366965	**6.047157017**				

**45**	**Transcription intermediary factor 1-beta**	**TIF1B_HUMAN**	**Q13263**	0.005	0.0151401	0.0435923	0.347311072	**2.879263237**	5.52-90261	**109**<br>2.6E^{-07}	11/22	16
**46**				0.002	0.0122145	0.0421808	0.289574878	**3.453338246**				
**47**				0.027	0.0190551	0.0476007	0.40031134	**2.498055639**				
**48**	**Heterogeneous nuclear ribonucleoprotein U**	**HNRPU_HUMAN**	**Q00839**	0.004	0.0217492	0.0640288	0.339679092	**2.943955113**	5.76-91269	**60**<br>8.5E^{-04}	7/14	10
**49**	**Heterogeneous nuclear ribonucleoprotein U**	**HNRPU_HUMAN**	**Q00839**	0.000	0.0198534	0.0624223	0.318049435	**3.144165305**	5.76-91269	**110**<br>2E^{-07}	8/9	13
**50**	**Heterogeneous nuclear ribonucleoprotein U**	**HNRPU_HUMAN**	**Q00839**	0.027	0.0166104	0.043295	0.383655541	**2.606504773**	5.76-91269	**70**<br>1.9E^{-03}	5/5	9
**51**	**DNA replication licensing factor MCM2**	**MCM2_HUMAN**	**P49736**	0.029	0.0012638	0.0041363	0.305540499	**3.272888545**	5.34-102516	**165**<br>6.4E^{-13}	17/27	25
**52**				0.039	0.0012606	0.0041951	0.300503048	**3.327753268**				
**53**	**Hypoxia up-regulated protein 1**	**HYOU1_HUMAN**	**Q9Y4L1**	0.029	0.0021725	0.0067587	0.321432887	**3.111069342**	5.16-111494	**101**<br>1.6E^{-06}	8/8	10
**54**				0.039	0.0014627	0.0058263	0.25104775	**3.983305971**				
**55**				0.015	0.0065333	0.0216408	0.301897653	**3.312380835**				
**56**				0.009	0.0043338	0.0135865	0.318976634	**3.135025874**				
**57**				0.001	0.0459592	0.0153109	**3.001739505**	0.333140167				
**58**				0.048	0.001202	0.0048902	0.245797664	**4.068386919**				
**59**	**Eukaryotic translation initiation factor 3 subunit B**	**EIF3B_HUMAN**	**P55884**	0.026	0.0161619	0.0323538	0.499537406	**2.001852089**	4.89-92823	**153**<br>1E^{-11}	14/18	20
**60**				0.040	0.0165049	0.0848576	0.194500986	**5.141362117**				
**61**	**Alpha-enolase**	**ENOA_HUMAN**	**P06733**	0.007	0.0073665	0.0307339	0.23968621	**4.172121541**	7.01 - 47481	**197**	15/20	32

										4.1E^{-16}		
**62**	**Isocitrate dehydrogenase [NAD] subunit alpha. mitochondrial**	**IDH3A_HUMAN**	**P50213**	0.004	0.0408057	0.0139044	**2.934736121**	0.340746138	6.47 - 40022	**93** 9.8E^{-06}	7/12	19
**63**	**Ribose-phosphate pyrophosphokinase 1**	**PRPS1_HUMAN**	**P60891**	0.001	0.0350293	0.0925712	0.378404227	**2.642676614**	6.51-35325	**152** 1.3E^{-11}	10/14	35
